# A study of the impact of social participation on the subjective well-being of low-vision older adults in Western China in the context of healthy aging

**DOI:** 10.1186/s12889-025-25914-z

**Published:** 2026-01-03

**Authors:** Zhu Zhong, Xu Luo, Jinyu Huang, Yajiang Li, Yuting He, Yu Luo

**Affiliations:** 1 Department of Teaching and Research, Jiangbei Campus of The First Affiliated Hospital of Army Medical University (The 958th Hospital of Chinese People’s Liberation Army), Chongqing, China; 2 School of Nursing, Army Medical University, Chongqing, China

**Keywords:** Low vision, Subjective well-being, Social participation, The elderly, Social support, Vision-related quality of life

## Abstract

**Background:**

Low vision represents a significant global health issue, and its effects on the physical and mental health of the elderly have been extensively researched. However, the relationship between social participation and the subjective well-being of elderly individuals with low vision in China remains underexplored. This study focuses on the elderly with low vision residing in the main urban area of Chongqing, Western China, to investigate the impact and pathways of subjective well-being at both subjective and objective levels of social participation.

**Methods:**

Purposive and convenience sampling methods were employed to select 274 elderly individuals with low vision who were monitored at the ophthalmology clinics of two tertiary hospitals in Chongqing, western China, as the subjects of this survey. The study utilized the Memorial University of Newfoundland Happiness Scale, the Social Participation Scale, and the Social Support Scale, along with a visual function-related quality of life scale to collect relevant data. The Social Participation Scale consists of two components: social participation activities（positive scoring) and subjective feelings of social participation (reverse scoring).Pearson correlation analysis was conducted to examine the relationships among the research variables. Based on the theoretical model, a multiple mediating effect prediction model was established to assess the impact of social participation on subjective well-being. Linear regression analysis and the Bootstrap method were applied to evaluate the mediating effects within the prediction model.

**Results:**

The overall average subjective well-being of elderly individuals with low vision in western China is measured at 24.75±8.02 points, which indicates a lower-middle level of well-being. participation in social activities is a positive predictor of subjective well-being, whereas individuals' subjective feelings of social participation can negatively impact it. social support and quality of life related to visual functioning were identified as mediating factors influencing the relationships between social participation activities, feelings of social participation, and subjective well-being.

**Conclusion:**

This study highlights the subjective well-being of elderly individuals with low vision in western China, as well as the pathways influencing the relationship between social participation-both subjective and objective-and their subjective well-being. It is recommended that healthcare professionals prioritize long-term care strategies for elderly patients experiencing low vision.

## Background

Low vision is a type of visual impairment that remains unresponsive to treatments such as medication, standard optometric correction, or surgery [1]. While the age-standardized prevalence of low vision is currently declining on a global scale, the absolute number of individuals affected continues to rise, driven by factors such as population growth and aging [2]. The World Health Organization (WHO) estimates that approximately 253 million people worldwide experience visual impairment, with about two-thirds of this demographic being over 60 years old [3]. In China, the prevalence of visual impairment among middle-aged and elderly individuals is reported to be 6.22% [4]. Age has been identified as a primary risk factor for the development of low vision [1]. As the aging of the Chinesepopulation continues to intensify, the proportion of elderly individuals with low vision is projected to increase further [5].

In older adults with low vision, restricted mobility can lead to altered social participation behaviors, characterized by increased time spent in isolation and decreased social interactions [6]. Moreover, persistent or progressive vision loss amplifies the fear of further impairment within this demographic, which is associated with a heightened risk of developing anxiety and depression [7]. Additionally, the negative emotions arising from vision loss may deter older adults from adopting adaptive strategies intended to mitigate the effects of their impairment [8]. This avoidance exacerbates the impact of negative emotions, making it even more difficult to take action to seek measuresto address low vision impairment, and improve the Vision Related Quality of Life(VRQOL) [8]. It is clear that the repercussions of low vision in older adults extend beyond physical health, encompassing significant implications for social functioning and mental well-being.

A substantial body of research has established that social participation positively impacts subjective well-being in older adults. However, the exploration of this relationship among older adults with low vision has received comparatively little attention from Chinese scholars. Previous studies have primarily examined social participation activities as a mediating variable, investigating how these activities may influence the subjective well-being of older adults with low vision through both moderating and mediating mechanisms. In light of the World Health Organization’s definition of Healthy Ageing (HA) as “the process of developing and maintaining the functional capacity of older people to achieve a sense of well-being” [9], this study aims to investigate the effects and pathways of social participation on subjective well-being, specifically focusing on older adults with low vision.

## Methoods

### Participants and procedure

This study employed a cross-sectional survey design utilizing both convenience and purposive sampling methods. The target population consisted of older adults with low vision who were followed up at the ophthalmology clinics of two tertiary-level hospitals in Chongqing, Western China, from October 2020 to March 2021. According to the estimation requirements of sample size for cross-sectional surveys, that is, the sample size should be 5–10 times the number of scale items [10], the scale with the largest number of items in this study is the social participation scale, with a total of 46 items, according to the number of items 5–10 times the estimate, and considering the dropout of the sample size, it was expanded by 10%, and the sample size was estimated to be at least 253 cases. Ultimately, 277 questionnaires were distributed; however, three participants withdrew during the survey, leading to a final count of 274 valid responses.

The inclusion criteria for the study population were as follows: (1) individuals aged 65 years or older; (2) residents of the main urban area of Chongqing City for at least one year; (3) a clear diagnosis of visual impairment in accordance with the 2019 WHO Visual Impairment Classification Criteria [1], defined as a Presenting Visual Acuity (PVA) in the better eye of less than 0.5 but greater than 0.05); (4) diagnoses related to age-related visual impairment, including macular degeneration, glaucoma, retinal diseases, optic nerve diseases, high myopia fundopathy, and failure to improve visual function following cataract surgery; (5) presence of eye diseases that cannot be treated or corrected through refraction, surgery, or medication; and (6) voluntary participation in the study after a detailed explanation of the informed consent form.

The exclusion criteria included: (1) individuals with severe hearing impairment or concurrent psychological disorders; (2) those with other serious systemic diseases; (3) individuals who required eye surgery in the near future; (4) those in the surgical or terminal stages of illness; and (5) individuals unable to communicate effectively, which hindered their ability to cooperate with the completion of the survey.

Prior to administering the questionnaire survey, the head nurse of the department was contacted to obtain permission and support. The purpose, content, and significance of the study were clearly explained to the respondents who met the criteria for nativity. The questionnaire was administered in the study room of the ophthalmology outpatient clinic after securing their consent. Given that the respondents were elderly individuals with low vision, which may hinder their ability to read the questionnaire entries, a one-on-one, question-by-question interview format was employed to collect the data. The researcher maintained a neutral tone of voice while reading the questions and accurately recording the responses. This study received approval from the Ethics Committee of the Army Medical University (approval number 2020-013>-02). The datasets in the current study are not publicly available due to confdentiality and security as they include sensitive private information, but are available from the corresponding author on reasonable request.

### Measurements

#### Sociodemographic characteristics

The socio-demographic characteristics of this study included six dimensions such as gender, literacy, mode of residence, marital status, economic income, and presence of chronic diseases.

#### Memorial university of Newfoundland scale of happiness (MUNSH)

This scale was developed by Kozma and Stones, and subsequently revised into Chinese by scholars Rengang Liu and others. The revision preserved the original meaning of the scale while adapting it to the Chinese cultural context and the living habits of the elderly [11]. At present, it is a more commonly used scale to evaluate the subjective well-being of the elderly. The MUNSH serving as a comprehensive reflection of both the short-term emotional responses and long-term emotional experiences of the study participants across four dimensions: positive affect (PA), negative affect (NA), positive experience (PE), and negative experience (NE) [12]. Scores of 36 or higher indicate a higher level of subjective well-being (SWB), scores of 12 or lower indicate a low level of SWB, while scores falling between these two thresholds represent a moderate level of SWB [12].

#### Community participation index (CPI)

This scale was developed and compiled by a team led by the American scholar Heinemann [13] and focuses on individuals’ self-evaluation of the frequency of social participation activities and their subjective feelings of social participation. Social participation activities were categorized into three main groups: family activities, social and recreational activities, and work role activities. Which are assessed using a positive scoring method, A score of 0 was assigned for ‘no’ participation, with higher scores reflecting increased frequency of participation. The subjective feelings of social participation encompass perceived importance and controllability. Utilizing a reverse scoring method, a higher scores indicate that individuals perceive or experience greater restrictions on social participation, which signifies a poorer overall experience of social participation [13].

#### Social support rating scale (SSRS)

The questionnaire quantitatively assesses an individual’s level of social support across three dimensions: objective support, subjective support, and the utilization of support. It was developed by the Chinese scholar Xiaoand demonstrates strong reliability and validity, making it widely utilized in China. The score ranges from 12 to 66 points, with higher scores indicating a greater level of social support [14].

#### National eye Institute visual function questionnaire (NEI-VFQ-25)

This questionnaire was compiled and developed by the National Eye Institute of the United States [15]. It serves as a multi-dimensional assessment of the ability to perform daily activities related to vision and is frequently recommended for use in research involving the elderly population [16].The questionnaire comprises 25 self-reported items that encompass 12 dimensions. Higher scores reflect a better quality of life associated with visual function [17]. Due to the fact that individuals with low vision cannot drive [18], the item regarding driving was excluded from the questionnaire.

### Data analysis

Data entry and collation were performed using Epidata 3.1 and Excel software, while the questionnaire data were analyzed with SPSS version 23.0 after thorough verification. The demographic characteristics, subjective well-being, frequency of socially engaged activities, and feelings of social engagement among respondents were statistically analyzed using means and standard deviations. To examine differences in subjective well-being among older adults with low vision across various demographic control variables, t-tests and one-way ANOVA were employed. Pearson correlation analysis was conducted to assess the relationships between social participation activities, subjective feelings of social participation, and each of the study variables individually. Additionally, multiple mediation effects modeling was utilized, grounded in theoretical frameworks regarding the impact of social support and quality of life related to visual function on health outcomes. After controlling for statistically significant demographic variables (*p* < 0.05), linear regression analysis was performed to evaluate the validity of the predictive model. The bootstrap method was applied to assess the mediating effects, with significance determined when the 95% confidence interval excluded zero. This analysis was executed using SPSS PROCESS macro version 3.0.

## Results

### Subjective well-being of older people with low vision

In this study, the overall mean score for subjective well-being (SWB) among older adults with low vision was 24.75 ± 8.02 points, which is significantly lower than the Chinese norm [19] (Table 1). Notably, 83.21% of participants reported a medium level of subjective well-being, with a mean score of 25.31 ± 6.25 points. Additionally, 6.93% of participants exhibited a higher level of subjective well-being, while 9.85% of participants were classified as having a lower level of subjective well-being.

**Table 1:** Comparison of subjective well-being scores of older adults with low vision to the norm (*n* = 274)

Groups	TotalSWB score	Positive emotion	Positive experience	Negative emotion	Negative experience
Older people with low vision	24.75 ± 8.02	4.70 ± 2.50	6.20 ± 2 0.90	3.52 ± 1.74	6.64 ± 2.13
normality	28.70 ± 10.72	4.49 ± 2.99	2.66 ± 2.71	7.24 ± 3.98	4.31 ± 3.38
t	−8.154>	1.420	20.255	−35.366>	18.063
*p*	< 0.001	0.157	< 0.001	< 0.001	< 0.001

### Analysis of differences in demographic characteristics of subjective well-being among older adults with low vision

The mean age of the subjects in this study was 73.70 ± 6.56 years. The Subjective well-being of elderly individuals with low vision varies significantly across different levels of education (*p* < 0.05), marital status (*p* < 0.01), monthly income per capita (*p* < 0.001), and the presence of other chronic diseases (*p* < 0.001). These differences were statistically significant(Table 2).This suggests that the aforementioned four demographic variables are influential factors affecting the subjective well-being of elderly individuals with low vision.

**Table 2:** Differences in SWB on demographic factors in older adults with low vision (*n*=274)

Item	Category		n	SWB Mean (SD)	Level of SWB (Number of cases/percentage)			F or t	*P* value
					Lower	Moderate	High		
Gender	male		126	25.01±8.25	13 (4.7)	103 (37.6)	10 (3.6)	0.485	0.628
	female		148	24.53±7.83	14 (5.1)	125 (45.6)	9 (3.3)		
Education level	Primary school or below		123	24.37±7.68	12 (4.4)	104 (38.0)	7 (2.6)	2.955	0.033
	junior high school		85	23.92±8.22	13 (4.7)	67 (24.5)	5 (1.8)		
	Senior high school		32	24.44±7.87	1 (0.4)	29 (10.6)	1 (0.7)		
	College and above		34	28.50±8.16	1 (0.4)	28 (10.2)	2 (0.7)		
Living arrangement	Live with a spouse only		148	24.82±7.96	14 (5.1)	123 (44.9)	11 (4.0)	1.288	0.275
	Live with children/grandchildren only		40	22.78±8.66	5 (1.8)	33 (12.0)	2 (0.7)		
	Live With spouse and children		61	26.30±7.35	3 (1.1)	53 (19.3)	5 (1.8)		
	Live to an institution for the elderly		6	23.17±6.65	1 (0.4)	5 (1.8)	0 (0.0)		
	live alone		19	23.95±9.17	4 (1.5)	14 (5.1)	1 (0.4)		
Marital status	unmarried		1	32.00±0.00	0 (0.0)	1 (0.4)	0 (0.0)	3.982	0.008
	married		212	25.27±7.75	17 (6.2)	179 (65.3)	16 (5.8)		
	divorcee		11	28.36±8.43	1 (0.4)	8 (2.9)	2 (0.7)		
	bereaved of one's spouse		50	21.62±8.34	9 (3.3)	40 (14.6)	1 (0.4)		
Monthly household income per capita (RMB)	<1000		18	20.94±5.80	2 (0.7)	16 (5.8)	0 (0.0)	12.456	<0.001
	1000-2999		122	22.23±7.82	20 (7.3)	98 (35.8)	4 (1.5)		
	3000-4999		98	27.23±7.51	5 (1.8)	82 (29.9)	11 (4.0)		
	≥5000		36	28.44±7.48	0 (0.0)	32 (11.7)	4 (1.5)		
Other chronic disease	Yes		180	23.27±8.03	22 (8.0)	147 (53.6)	11 (4.0)	4.366	<0.001
	No		94	27.59±7.22	5 (1.8)	70 (25.5)	19 (6.9)		
Independent variable		minimum value	maximum values		Mean ± standard deviation		Average score per entry/dimension		
Social participation activities		2	39		17.30±7.15		0.87±0.36		
Feelings of social participation		66	134		111.16±17.93		4.12±0.66		
Social support		15	42		25.53±7.45		2.55±0.75		
Visual function-related quality of life score		24	66		45.55±8.05		3.79±0.67		

### Correlation analysis of research variables

Mean values of social participation activities are 17.30 ± 7.15, the subjective feelings of social participationl are measured at 111.16 ± 17.93, while social support is recorded at 25.53 ± 7.45. Additionally, the visual-related quality of life is assessed at 45.55 ± 8.05.The results of the correlation analysis indicated that social participation activities exhibited moderate positive correlations with subjective well-being (*r* = 0.695, *p* < 0.01), social support (*r* = 0.740, *p* < 0.01), and quality of life related to visual functioning (*r* = 0.604, *p* < 0.01); Conversely, feelings of social participation were found to have moderate negative correlations with subjective well-being (*r*=- 0.809, *p* < 0.01), social support (*r*=−0.885>, *p* < 0.01), and quality of life related to visual functioning (*r* = −0.674>, *p* < 0.01). These correlations provide empirical support for the proposed mediation effect model.(Table 3).

**Table 3:** Correlation analysis of social participation activities, social support, VRQOL and subjective well-being (r value, *n* = 274)

Variables	Social participation activities	The subjective feelings of social participation	Social support	VRQOL	SWB
Social participation activities	1				
The subjective feelings of social participation	−0.696>**	1			
Social support	0.740**	−0.885>**	1		
VRQOL	0.604**	−0.674>**	0.647**	1	
SWB	0.695**	−0.809>**	0.818**	0.731**	1

### Impact of social participation on subjective well-being

This study explores predictive modeling of social support-mediated mediation effects, utilizing convoy modeling theory to examine the impact of social support on health [20]. The predictive model specifically addresses the mediating effect of visual function-related quality of life, drawing upon Lazarus and Folkman’s stress response model [18] as a foundational framework.

#### Relationship between the effects of social participation activities and subjective well-being

**Fig. 1:**
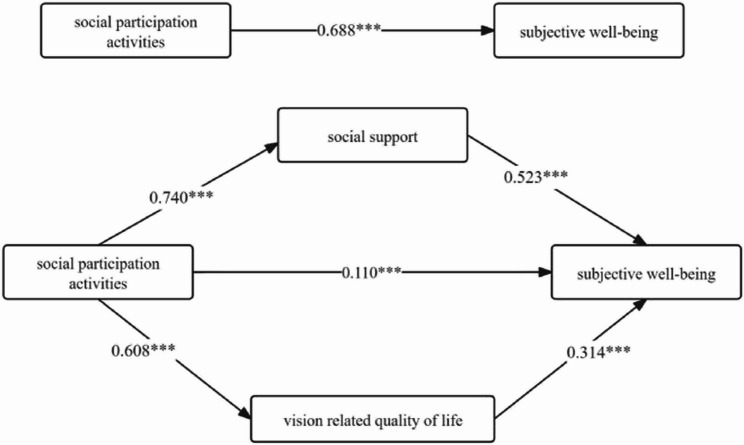
Social support, VRQOL-mediated social engagement activities and subjective well-being model and path coefficients. Note: Path coefficients are standardized coefficients

The regression analysis conducted between the variables indicated that the overall effect of social engagement activities on subjective well-being was significant, with coefficients of B = 0.771, t = 13.418, and *p* < 0.001. Specifically, both social support (B = 0.563, t = 10.494, *p* < 0.001) and visual function-related quality of life (B = 0.313, t = 7.487, *p* < 0.001) emerged as significant positive predictors of subjective well-being. The results from the Bootstrap method used to test for mediating effects demonstrated that social support (Boot 95%CI = 0.339 to 0.542) and visual function-related quality of life (Boot 95%CI = 0.150 to 0.287) significantly mediated the relationship between social participation activities and subjective well-being, with effect sizes of 56.29% and 27.89%, respectively(refer to Table 4; Fig. 1).

**Table 4:** Social Support, VRQOL-Mediated social participation Activities, and subjective Well-Being model effect values by path (*n* = 274)

	Efficiency value	Boot SE	Boot LLCI	Boot ULCI	Counterpart efficiency value
Aggregate effect	0.771	0.056	0.665	0.888	-
Direct effect	0.123	0.051	0.023	0.219	15.95%
Total indirect effect	0.649	0.060	0.537	0.772	84.18%
Indirect effect 1: Social participation activities→ SSRS → SWB	0.434	0.052	0.339	0.542	56.29%
Indirect effect 2: Social participation activities →VRQOL→SWB	0.215	0.035	0.150	0.287	27.89%

#### Effect relationship between feelings of social participation and subjective well-being

**Fig. 2:**
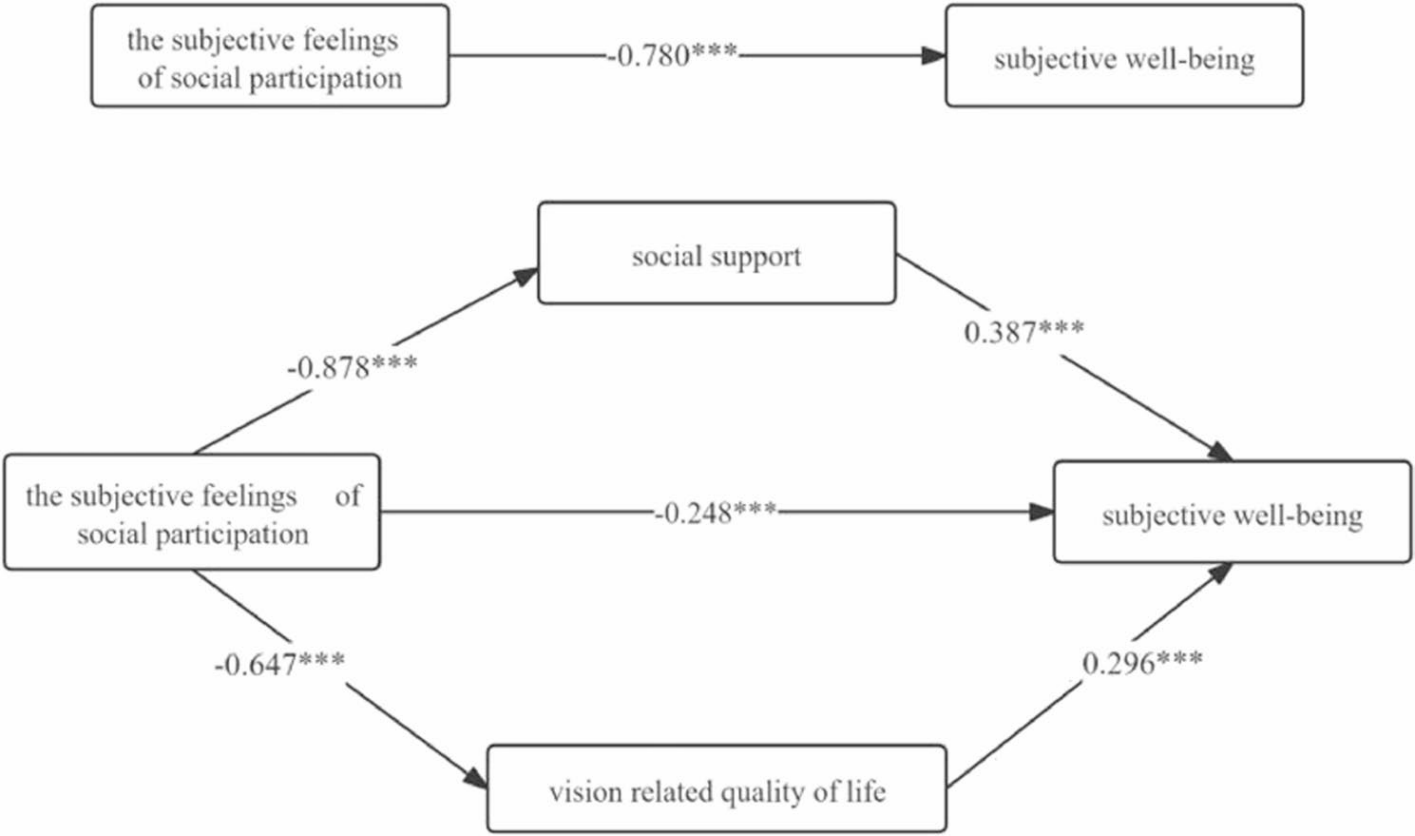
Social support, VRQOL-mediated model of perceived social participation and subjective well-being and path coefficients. Note: Path coefficients are standardized coefficients

Regression analysis revealed a significant total effect of feelings of social participation on subjective well-being (B = −0.349>, t = −20.058>, *p* < 0.001).Furthermore, the direct effect of feelings of social participation on subjective well-being remained significant after controlling for social support and quality of life related to visual functioning (B = −0.111>, t = −3.450>, *p* < 0.01).social support (B = 0.417, t = 5.584, *p* < 0.001) and quality of life related to visual functioning (B = 0.295, t = 7.074, *p* < 0.001) emerged as significant positive predictors of subjective well-being. The bootstrap method for testing mediating effects indicated that social support (Boot 95% CI = −0.202> to −0.100>) and quality of life related to visual functioning (Boot 95% CI = −0.114> to −0.060>) had significant mediating effects between feelings of social participation and subjective well-being, with effect sizes of 43.55% and 24.64%, respectively (Table 5; Fig. 2).

**Table 5:** Social Support, VRQOL-Mediated feelings of social participation and subjective Well-Being model effect values by path (*n* = 274)

	Efficiency value	Boot SE	Boot LLCI	Boot ULCI	Oppose efficiency value
Aggregate effect	−0.349>	0.017	−0.382>	−0.314>	-
Direct effect	−0.111>	0.030	−0.173>	−0.055>	31.81%
Total indirect effect	−0.238>	0.027	−0.290>	−0.184>	68.19%
Indirect effect 1: Feelings of social participation → SSRS → SWB	−0.152>	0.026	−0.202>	−0.100>	43.55%
Indirect effect 2: Feelings of social participation → VRQOL → SWB	−0.086>	0.014	−0.114>	−0.060>	24.64%

## Discussion

To the best of our knowledge, this study represents the first comprehensive exploration of the effects and pathways through which social participation influences subjective well-being, examining both objective and subjective dimensions. It assesses the mechanisms linking social participation to subjective well-being specifically in older adults with low vision, offering a more nuanced analysis than previous research. The findings of this study not only broaden the understanding of subjective well-being within the aging population but also provide a reliable and detailed foundation for community health workers to develop and implement health care strategies tailored to older adults with low vision.

### Current status of subjective well-being among older adults with low vision

The findings of this study indicate that the subjective well-being of older adults with low vision falls within the lower middle range, contrasting with the results of previous research [21–23]. Earlier studies have demonstrated a correlation between low vision and reduced levels of subjective well-being. The discrepancy in findings may be attributed to factors such as emotional adaptation and the use of varying evaluative indicators.Schilling et al. [24] investigated the evolution of positive and negative emotions over time in older adults with age-related macular degeneration. They discovered that positive emotions exhibited a significant and nonlinear relationship with the duration of the disease; specifically, positive emotions were markedly lower during the initial stages of vision loss, gradually increased over time, and then stabilized after reaching a certain duration. Adaptation theory posits that, despite experiencing a decline in physical functioning, older adults’ subjective well-being tends to stabilize over time [25].Previous research on the theories of adaptation in late-life happiness has found that negative affective influences on well-being are more pronounced when specific measures related to visual functioning are employed to assess subjective well-being. In contrast, the differences in effect sizes for well-being components are less pronounced when emotional experience is utilized as an assessment tool for subjective well-being in older adults with low vision [26].The Memorial University of Newfoundland Happiness Scale, selected for this study, primarily evaluates subjective well-being through the lenses of emotional experience and life satisfaction, which may explain the deviation from the findings of earlier studies.

### The effect of social participation activities on the subjective well-being of older adults with low vision

The analysis of the impact relationship model developed in this study revealed that social participation activities positively predict the subjective well-being of older adults with low vision, aligning with findings from previous studies [27, 28]. Furthermore, the mediation effect model suggests that the social support and the VRQOL mediate the relationship between socially engaged activities and subjective well-being. The theoretical framework regarding the impact of social support on health posits that social support serves as a buffer during stressful events, thereby safeguarding overall health [29]. Gong et al. [27] examined the potential connection between social support and depression in older adults with visual impairment, demonstrating that positive social support correlates with a reduced risk of depression in this population. Paul et al. [30]investigated older adults with sensory impairments and found that diminished social support was linked to heightened feelings of loneliness among older adults with visual impairment. the stress coping model posits that low vision constitutes a stressful event in the lives of older adults, with visual function-related quality of life being influenced by coping strategies [31].Research has demonstrated that adaptive coping strategies, such as orientation and mobility training, enhance the independence of older adults with low vision in daily activities, thereby reducing limitations and fostering greater social participation [32].A survey conducted by Xu et al. [33] on patients with age-related macular degeneration revealed that appropriate support and low-vision rehabilitation significantly improved visual function-related mobility, thereby helping to maintain a satisfactory quality of life.In summary, social support exerts a buffering effect on negative emotions in older adults with low vision. While visual function-related quality of life is a modifiable factor intricately linked to functional abilities for social participation and serves as a crucial determinant of emotional well-being [31]. The findings of this study indicate that the relationship between social support and visual function-related quality of life significantly influences subjective well-being. This path is consistent with prior research. The results of this study can provide new insights for healthcare professionals when implementing community health promotion programs for elderly individuals with low vision. By expanding the types of participation based on individual needs for social engagement, initiatives such as interest groups tailored to their participation can be developed.

### The effect of feelings of social participation on subjective well-being

The subjective feelings of social participation is a subjective experience that reflects the perceived or experienced limitations individuals face in engaging socially [34]. This subjective feelings arises from the dynamic interaction between the individual and their environment [34]. The biopsychosocial model serves as a theoretical framework to explain these subjective feelings. Environmental factors in social interactions, such as perceived or experienced public discrimination against low vision, affect the social participation feelings of low vision elderly people, such as social exclusion, marginalization, etc., which will cause low vision elderly people to give up opportunities for social participation [35], In China, elderly individuals with low vision may experience limited participation due to insufficient social environmental support for engagement [36]. while reduced social participation is associated with increased social isolation and loneliness [37]. The above research results can explain the negative impact of social participation feelings on the subjective well-being of low-vision elderly people in the results of this study. The results of the path analysis prediction model established in this study show that social support and VRQOL play a partial mediating role between feelings of social participation and subjective well-being. A relevant theoretical model can elucidate the findings of this study, The public’s stereotype regarding low vision reflects the social participation pressures experienced by elderly individuals with low vision, where social support can serve a useful buffering role during stressful events [38]. The limited independence and mobility of elderly individuals with low vision act as barriers to social participation. When they perceive challenges related to their visual function as uncontrollable, they may resort to avoidant coping strategies. Research indicates that negative coping strategies, such as avoidance, hinder the maintenance and enhancement of both independence and mobility, thereby adversely affecting the quality of life associated with visual function [39]. This, in turn, can lead to an increase in negative emotion [31]. Future community nursing should consider developing rehabilitation and continuity care measures based on the subjective experiences of social participation among elderly individuals with low vision. This could include guiding them in learning to use low-vision assistive devices and identifying and mitigating mobility and environmental risks that hinder their participation.

This study provides recommendations for healthcare professionals and community health workers when implementing relevant care programs. It considers the adoption of a family-centered education model to guide family members in assisting visually impaired elderly individuals to adapt to changes in daily living skills that are visually guided. The application of existing visual functions aims to maintain independence and, in turn, promote social participation. Furthermore, it assists communities in providing social participation platforms for visually impaired elderly individuals. Based on the needs of these individuals regarding social participation, intelligent technologies such as voice calling and accessible voice conversion can be utilized to create various forms of social participation platforms. Additionally, it encourages relevant public health service departments to create an accessible social participation environment for visually impaired elderly individuals, such as considering the enhancement of safety facilities for public transportation and ensuring the ground surface in participation environments is level, thereby minimizing the environmental barriers faced by visually impaired elderly individuals in social participation.

### Limitations and future research

This study has several limitations. Firstly, Due to constraints in time, energy, and resources, a convenience sampling method was employed to conduct a cross-sectional survey at only two tertiary hospitals in Chongqing City. Consequently, the sample size was limited, and the findings may not accurately represent the overall population of older adults with low vision in China, thereby affecting the generalizability of the results. Future research should aim to expand the sample size and geographic scope, employing stratified and random sampling techniques to enhance sample representativeness. Secondly, The survey tool used in this study is of a self-reporting nature and employs a singular method to assess subjective well-being, which may be subject to self-report bias. Future studies should consider incorporating multiple indicators related to subjective well-being, such as quality of life and depression, to provide a more comprehensive assessment of the subjective well-being of older adults with low vision.

## Conclusions

This study revealed that the subjective well-being of older adults with low vision is positioned at a moderately low level. The multiple mediation effect model shows that social participation activities and subjectivefeelings of social participation, are related to the subjective well-being of elderly individuals with low vision.Additionally, social support and vision-related quality of life partially mediate the relationship between social participation activities, subjective feelings of social participation, and subjective well-being. This suggests that professional caregivers and community healthcare workers should focus on both the subjective and objective aspects of social participation, in addition to the perspective of ophthalmic diseases, when providing continuous care services for elderly individuals with low vision. In addition to encouraging individuals to adopt proactive measures to maintain their participation in social activities, such as utilizing visual assistive technologies, rehabilitation care measures aimed at improving visual functional abilities related to participation can also be developed from the perspective of subjective feelings of social engagement. These may include mobility training and self-management training, enabling individuals to achieve a sense of subjective well-being through their self-worth and recognition in social participation. Due to the mediating role of social support in the relationship between social participation and subjective well-being, governments and healthcare institutions should consider incorporating the needs of elderly individuals with low vision for emotional support and tools for social participation into their focus areas. This provides new insights for developing home care strategies and continuity of care measures.

## Data Availability

The datasets in the current study are not publicly available due to confdentiality and security as they include sensitive private information, but are available from the corresponding author on reasonable request.
